# Does anodal cerebellar tDCS boost transfer of after-effects from throwing to pointing during prism adaptation?

**DOI:** 10.3389/fpsyg.2022.909565

**Published:** 2022-09-27

**Authors:** Lisa Fleury, Francesco Panico, Alexandre Foncelle, Patrice Revol, Ludovic Delporte, Sophie Jacquin-Courtois, Christian Collet, Yves Rossetti

**Affiliations:** ^1^INSERM UMR-S, CNRS UMS, Trajectoires Lyon Neuroscience Research Center (CRNL), Bron, France; ^2^Defitech Chair for Clinical Neuroengineering, Center for Neuroprosthetics (CNP) and Brain Mind Institute (BMI), École polytechnique fédérale de Lausanne (EPFL) Valais, Sion, Switzerland; ^3^Department of Psychology, University of Campania “Luigi Vanvitelli”, Caserta, Italy; ^4^“Mouvement et Handicap” Platform, Neurological Hospital, Hospices Civils de Lyon, Bron, France; ^5^Inter-University Laboratory of Human Movement Biology, Villeurbanne, France

**Keywords:** prism adaptation (PA), transfer, cerebellum, anodal tDCS, sensorimotor plasticity

## Abstract

Prism Adaptation (PA) is a useful method to study the mechanisms of sensorimotor adaptation. After-effects following adaptation to the prismatic deviation constitute the probe that adaptive mechanisms occurred, and current evidence suggests an involvement of the cerebellum at this level. Whether after-effects are transferable to another task is of great interest both for understanding the nature of sensorimotor transformations and for clinical purposes. However, the processes of transfer and their underlying neural substrates remain poorly understood. Transfer from throwing to pointing is known to occur only in individuals who had previously reached a good level of expertise in throwing (e.g., dart players), not in novices. The aim of this study was to ascertain whether anodal stimulation of the cerebellum could boost after-effects transfer from throwing to pointing in novice participants. Healthy participants received anodal or sham transcranial direction current stimulation (tDCS) of the right cerebellum during a PA procedure involving a throwing task and were tested for transfer on a pointing task. Terminal errors and kinematic parameters were in the dependent variables for statistical analyses. Results showed that active stimulation had no significant beneficial effects on error reduction or throwing after-effects. Moreover, the overall magnitude of transfer to pointing did not change. Interestingly, we found a significant effect of the stimulation on the longitudinal evolution of pointing errors and on pointing kinematic parameters during transfer assessment. These results provide new insights on the implication of the cerebellum in transfer and on the possibility to use anodal tDCS to enhance cerebellar contribution during PA in further investigations. From a network approach, we suggest that cerebellum is part of a more complex circuitry responsible for the development of transfer which is likely embracing the primary motor cortex due to its role in motor memories consolidation. This paves the way for further work entailing multiple-sites stimulation to explore the role of M1-cerebellum dynamic interplay in transfer.

## Introduction

The central nervous system acts as the conductor of over 600 muscles to produce an infinity of smooth and precise movements in everyday life. Sensorimotor adaptation is an error-driven process that enables to modify a movement in response to a perturbation (Bastian, 2008; Roemmich and Bastian, 2018; Prablanc et al., 2019).

Several paradigms enable to study sensorimotor adaptation in a laboratory context, among them one of the most studied is prism adaptation (PA; Welch, 1974; Redding et al., 2005). In a classical PA protocol, individuals wear prismatic goggles eliciting a lateral visual shift. When pointing at a target, they initially experience errors in the same direction of the prismatic shift (terminal errors). By repetition of the pointing movement, subjects rapidly regain their baseline accuracy. However, when prisms are removed, they show consistent after-effects, i.e., errors in the direction opposite to the prismatic shift (Redding et al., 2005; Petitet et al., 2017; Prablanc et al., 2019). After-effects probes the deployment of sensorimotor adaptive processes during the exposure period to face the encountered perturbation (Redding et al., 2005; Prablanc et al., 2019). Importantly, PA-induced plasticity showed substantial benefits in alleviating neglect symptoms suggesting that after-effects might be beneficial in neurorehabilitation (Rossetti et al., 1998; Frassinetti et al., 2002; Làdavas et al., 2011).

Beyond basic after-effects, an important issue is whether they are specific to the situation in which the perturbation has been experienced or whether they can be observed in other contexts, such as a different task (Poggio and Bizzi, 2004; Fleury et al., 2020). This represents a crucial interest in the field of neurorehabilitation. Indeed, adaptation processes could be highly relevant for patients who suffer sensorimotor disorders provided that sensorimotor transformations set-up during rehabilitation sessions could also apply beyond the context in which they emerged, i.e., in other daily life situations (Roemmich and Bastian, 2018). PA procedures enable researchers to easily assess after-effects transfer by testing the individuals’ performance in a different context once the prismatic shift has been removed. For example, participants can perform a pointing task during prism exposure. Then, once the perturbation is removed, after-effects can be assessed both on the same task (i.e., pointing) and on a different task (e.g., throwing) to assess after-effects transfer (Alexander et al., 2013; Fleury et al., 2020).

The literature on the mechanisms and brain networks involved during PA includes a plethora of studies ranging from theoretical papers describing models of PA (Welch, 1974; Redding and Wallace, 2001; Redding et al., 2005; Petitet et al., 2017), to neurological lesion (Weiner et al., 1983; Baizer et al., 1999; Pisella et al., 2004, 2005; Newport and Jackson, 2006; Hanajima et al., 2015), neuroimaging (Clower et al., 1996; Danckert et al., 2008; Luauté et al., 2009; Chapman et al., 2010; Küper et al., 2014) and neurostimulation studies (Panico et al., 2016, 2018a,b, 2022; Fleury et al., 2021). Evidence from these studies converge toward two processes at work during PA: a rapid process of strategic control responsible for quick error reduction during the very first trials of exposure and a slower process of realignment accounting for complete correction of errors during exposure and after-effect development (Prablanc et al., 2019). As far as brain regions activated during PA are concerned, literature converges in considering a network including cerebello-parieto-motor areas as being crucially involved during PA, with a main contribution of the cerebellum in after-effect development (for reviews see Panico et al., 2020, 2021).

Non-invasive brain stimulations such as transcranial Direct Current Stimulation (tDCS) provide a unique opportunity to modulate the activity of targeted brain region to investigate how it would affect the behavior supposed to be related to that area. Therefore, it is particularly suitable for studying the neural bases of PA as it allows to study the direct link between human brain and behavior (Panico et al., 2020). The cerebellum is an interesting candidate for an efficient neurostimulation given its position below the skull (van Dun et al., 2017). Noteworthy, studies modeling the electrical field during cerebellar neuromodulation demonstrated that the highest electric field and current density are found underneath the stimulation electrode, irrespective of the return electrode (Parazzini et al., 2013; Rampersad et al., 2014; Batsikadze et al., 2019). In addition, the current mainly spreads through the posterior part of the cerebellum (Batsikadze et al., 2019) which interestingly contains both sensorimotor lobules (VIII) and higher-level order zones (e.g., VII, Crus I and II) with some connections to the parietal cortex (Sasaki et al., 1975; Guell and Schmahmann, 2020). Plus, previous neuro-imaging studies suggested that the posterior part of the cerebellum was involved in several processes during PA, including error correction and realignment (e.g., Chapman et al., 2010; Küper et al., 2014). In the framework of a cerebello-parietal network approach, cerebellar tDCS therefore represents a meaningful and efficient way to functionally study the role of the cerebellum in PA processes.

However, while the scientific literature have widely explored the mechanisms and brain areas related to error compensation and after-effect development during PA (see Panico et al., 2020 for a review), sparse knowledge is available on the mechanisms underlying the transfer of after-effects, which are still not perfectly understood (Fleury et al., 2020).

Considering the role of the cerebellum in the development of after-effects (Weiner et al., 1983; Pisella et al., 2005; Luauté et al., 2009; Küper et al., 2014; Panico et al., 2016), it is possible to hypothesize that it may also be involved in their transfer, as the processes at work during exposure may also determine whether after-effects could be transferred to another task. In a first behavioral study on after-effect transfer, Fleury et al. (2020) showed that transfer largely depended on the type of task practiced during exposure. Indeed, participants who practiced a pointing task during exposure showed substantial transfer to the throwing task while those who performed a throwing task under the prismatic shift did not demonstrate any transfer on the pointing task. Interestingly, experts in throwing (dart players) did instead show transfer from throwing to pointing. In a second study, Fleury et al. (2021) showed that cathodal cerebellar transcranial Direct Current Stimulation (tDCS) partially reduced transfer of after-effects from pointing to throwing in healthy participants. Taken together, results from these two studies question whether the processes of transfer could be boosted by facilitatory brain stimulation. In other sensorimotor adaptation paradigms, by using anodal cerebellar tDCS, Galea et al. (2011) reported faster adaptation to visuomotor rotation and Leow et al. (2017) also reported increased after-effects in open loop reaching trials following explicit removal of the visuomotor rotation, but no similar data are available in the PA literature.

The aim of the present study was to investigate the functional role of the cerebellum in the inter-task transfer of after-effects from throwing to pointing during PA by using anodal tDCS. Based on our previous findings on after-effects transfer (Fleury et al., 2020, 2021) and on previous stimulation studies on visuomotor adaptation (Galea et al., 2011; Panico et al., 2016, 2018a,b; Leow et al., 2017) we hypothesized that anodal cerebellar tDCS would have a boosting effect, promoting transfer from throwing to pointing in non-expert participants. Investigating the neural correlates of after-effects transfer would provide crucial information on the nature and locus of adaptive processes allowing individuals to face a perturbation during exposure, and relevant clues about the adjustment of internal representations following sensorimotor adaptation (Poggio and Bizzi, 2004; Redding and Wallace, 2006).

## Materials and methods

26^1^ healthy participants voluntarily took part in the study (15 females, 11 males, mean age = 24.8 ± 4.6) with a pre-post design to assess the effect of real tDCS compared to placebo stimulation on behavioral outcomes measured before, during, after and prism adaptation. All procedures were approved by an ethics committee from the Inserm (“CPP SUD-EST IV,” ID-RCB: 2010-A01180-39) and were consistent with relevant guidelines and regulations. We collected each participant’s written informed consent before starting the experiment and they were free to stop the experimental procedure at any time.

### Participants

All participants were right-handed with no neurological condition or orthopedic disorder. They were naïve of the PA paradigm and had normal or corrected to normal vision. We split participants into two groups, depending on the tDCS parameters used during the procedure. Participants in the experimental “A-tDCS” group (*n* = 15, 7 males and 8 females, mean age = 23.2 ± 4.8) received anodal cerebellar stimulation during PA, while participants in the control “SHAM” group (*n* = 11, 4 males and 7 females, mean age = 26.9 ± 4.3) received placebo cerebellar stimulation. All participants followed the same experimental procedure except for the tDCS parameters.

### Experimental paradigm

**Figure 1:**
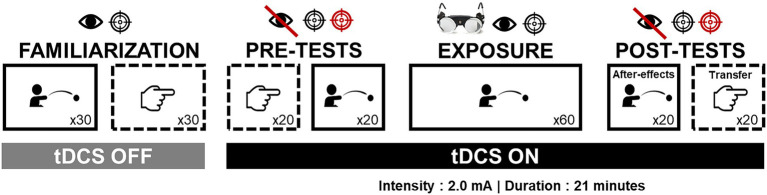
Experimental procedure. The figure depicts the different steps of the study and the conditions within each step, i.e., the task performed (throwing or pointing), visual feedback availability (bared eye or normal eye), trials number (i.e., 20, 30, or 60), the presence of prism goggles, targets number and color (one black target, or two targets—black and red), and tDCS conditions (Off or On). Solid boxes refer to the exposed task condition (i.e., throwing) while dotted boxes refer to the unexposed task condition (i.e., pointing).

The procedure was divided into four parts (familiarization, pre-tests, exposure, post-tests) as shown by Figure 1.

Depending on the stage of the procedure, the participants performed either pointing or throwing with or without visual control (closed-loop vs. open-loop conditions). They were requested to point at the central or alternatively at the central and the lateral target, depending on the stage (see Figure 1).

At the beginning of the procedure, participants sat in an adjustable and movable hospital chair, and remained seated throughout the entire experiment without being able to move the chair themselves. Participants wore eye patches during each transitional phase to prevent de-adaptation due to environmental vision after exposure. Participants were also instructed to remain motionless between each task. To limit the number of transitional phases, all participants performed the tasks in the same order (see Figure 1).

#### General procedures

Specific pointing and throwing conditions for each stage of the experimental protocol are detailed in the following section and illustrated in Figure 1. See also Fleury et al. (2021) for more detailed information about the task set-ups.

##### Familiarization

To familiarize with both tasks and with experimental settings, participants in both groups successively performed 30 trials of throwing and pointing. All were performed in closed-loop condition toward the central target.

##### Pre-tests

Pre-tests were designed to assess baseline performance. All participants performed 20 trials of pointing and throwing tasks. Trials were performed in open-loop condition and toward both targets in a pseudo-randomized order (the same for all participants).

##### Exposure

Participants performed 60 throwing trials while wearing the prismatic goggles laterally shifting the visual field by 10° to the right (OptiquePeter.com, Lyon). Trials were performed in closed-loop condition toward the central target, as fast and accurate as possible. While wearing the goggles, participants were asked to keep their eyes closed. They were also instructed not to look straight ahead (thus preventing to look at their own body) and to remain motionless before starting the experimental tasks while wearing the prismatic googles.

##### Post-tests

Once the prisms were removed, after-effects were first assessed during 20 throwing trials in open-loop condition toward both targets. Then, transfer was measured during 20 pointing trials under open-loop condition toward both targets.

### tDCS protocol

tDCS settings were based on the experimental designs used in previous studies (Panico et al., 2016; Fleury et al., 2021). A battery-driven stimulator (NeuroConn, neuroCare Group, Germany) delivered a constant current of 2 mA through a pair of surface saline-soaked sponge electrodes (area = 25 cm^2^).

Participants were timed to complete the experimental procedure within 21 min of stimulation as recommended by safety guidelines (Nitsche and Paulus, 2000; Woods et al., 2016). Participants who did not complete the procedure within this time range were excluded from the analysis. The anode was placed over the right cerebellum (1 cm below and 4 cm right to the inion) and the cathode was placed over the right deltoid muscle, to ensure selective stimulation over the right cerebellum. Stimulation was delivered over the right cerebellum, as all participants were right-handed (Schlerf et al., 2015).

During both sham and active conditions, the stimulation was gradually increased to 2 mA with a fade-in of 30 s. The sham stimulation was similarly performed as active stimulation, but the stimulation was active only during the fade-in (30 s) and immediately turned-off after a fade-out phase (30 s). This procedure ensured that participants felt the same itching sensation at the when starting tDCS as participants assigned to the experimental group, and were thus blind to the stimulation condition they had been assigned to (Gandiga et al., 2006).

### Data acquisition

We used an opto-electronic motion capture system (9 cameras, Vicon Motion Systems Ltd., Oxford; *Mouvement et Handicap* platform, *Hospices Civils* of Lyon) to track movements trajectories during pointing trials. We placed reflective markers on the index, the wrist and the elbow of each participant. For the throwing trials, reflective markers were placed on the throwing board to localize the targets and the projectiles were also reflective. This allowed us to record the ball impact on the vertical board for each throw.

### Data processing

#### Throwing and pointing terminal errors

For each trials, we filtered the recorded markers’ trajectories using a Butterworth low-band pass filter at a 6 Hz cut-off frequency. Then, the endpoint of each pointing movement was computed automatically using a customized software written in MATLAB®. Movement detection was ensured as following: we defined onset as the point at which hand velocity exceeded 80 mm/s while offset corresponded to the time-point at which velocity dropped below this cut-off (O’Shea et al., 2014). Following the automatic detection, we cross-checked visually all trials and adjusted the onset and offset if needed. We then computed the lateral endpoints errors between the index endpoint and the aimed target at movement offset for each trial.

Concerning the throwing trials, MATLAB customized routines allowed us to automatically detect the moment corresponding to the contact between the projectile and the board. We then obtained the lateral errors between the impact of the projectile and the aimed target. For both types of movements, we finally obtained performance on each trial, i.e., the angular deviation between the ball impact/index endpoint and the aimed target.

#### Pointing kinematics analysis

Pointing can be divided into two phases: the acceleration phase (initial ballistic component) and the deceleration phase, referring to the target approach phase (Elliott et al., 2010). The initial part of the trajectory reflects feedforward movement planning while the second part involves online feedback corrections (O’Shea et al., 2014). We analyzed these trajectories investigating the orientations of velocity vectors at acceleration, velocity and deceleration peaks. Orientations are defined as angles between the velocity vector and the line joining the starting position and the central target. Only movement kinematics during the pointing task were investigated, using a MATLAB in-house software written.

#### Analyses

We computed terminal errors between index endpoint (pointing task) or ball impact (throwing task) and the aimed target for each trial as a first dependent variable. The second dependent variable only concerned the pointing task and corresponded to the trajectories orientations as the magnitude of velocity vectors at acceleration peak (initial orientation), velocity peak (intermediate orientation) and deceleration peak (terminal orientation).

During pre-tests and post-tests, we computed terminal errors by grouping the right and central targets as a previous study using the same procedures reported no difference between targets (Fleury et al., 2020, 2021). We conducted a specific analysis of pointing trajectories for right and central targets as the initial directions and the length of trajectories were intrinsically dependent on the target position.

In addition, we subtracted pre-tests values for each individual and for each task from the post-tests values, as recommended in PA literature (Prablanc et al., 2019). In fact, quantifying after-effects requires to consider the individual physiological baseline deviation into account within the same group of testing.

We run linear mixed models (Singer and Willett, 2003) separately for each stage of the experiment and for each dependent variable. Therefore, we analyzed not only mean individuals’ values (e.g., mean of 20 post-tests trials) but also their evolution across time. In addition, linear mixed models are a flexible method appropriate to deal with intra-individuals’ variability within each group (Wright and London, 2009). We could thus assess inter-subject differences considering the intra-individual changes over time (through trial-by-trial repetition; Fleury et al., 2021).

During the different phases of the procedure, each trial was considered as a time point. All time points were the level-one unit nested in the different individuals (level-two units). Random intercept models tested the longitudinal effect of trials’ repetition (TIME factor) and the effect of the stimulation condition (GROUP factor: A-tDCS versus SHAM). The GROUP*TIME interaction assessed whether the slopes of the curves differed between groups. All analyses were conducted using R package labeled lme4 (Bates et al., 2015). A value of *p* of 0.05 was used to indicate statistical significance.

We also divided specific phases of the experiment into multiples series of trials, notably during exposure, to better specify longitudinal analysis of the variables. Indeed, PA literature describes several adaptive processes associated with different timing (fast vs. slow processes; Rossetti et al., 1993; Smith et al., 2006; Inoue et al., 2015; Petitet et al., 2017). Therefore, we analyzed early exposure (trials 1 to 10) separately from the trials 11 to 60 during exposure. We also analyzed separately each block of 10 trials under exposure to obtain a complete description of throwing under the prismatic perturbation, and to investigate the effect of tDCS at different times of exposure.

We performed independent samples *T*-tests to test the differences between mean group terminal errors and mean group trajectories orientation during familiarization. As no stimulation and no prismatic deviation were applied during familiarization, we considered no reason to test for any longitudinal variation across trials.

## Results

**Figure 2:**
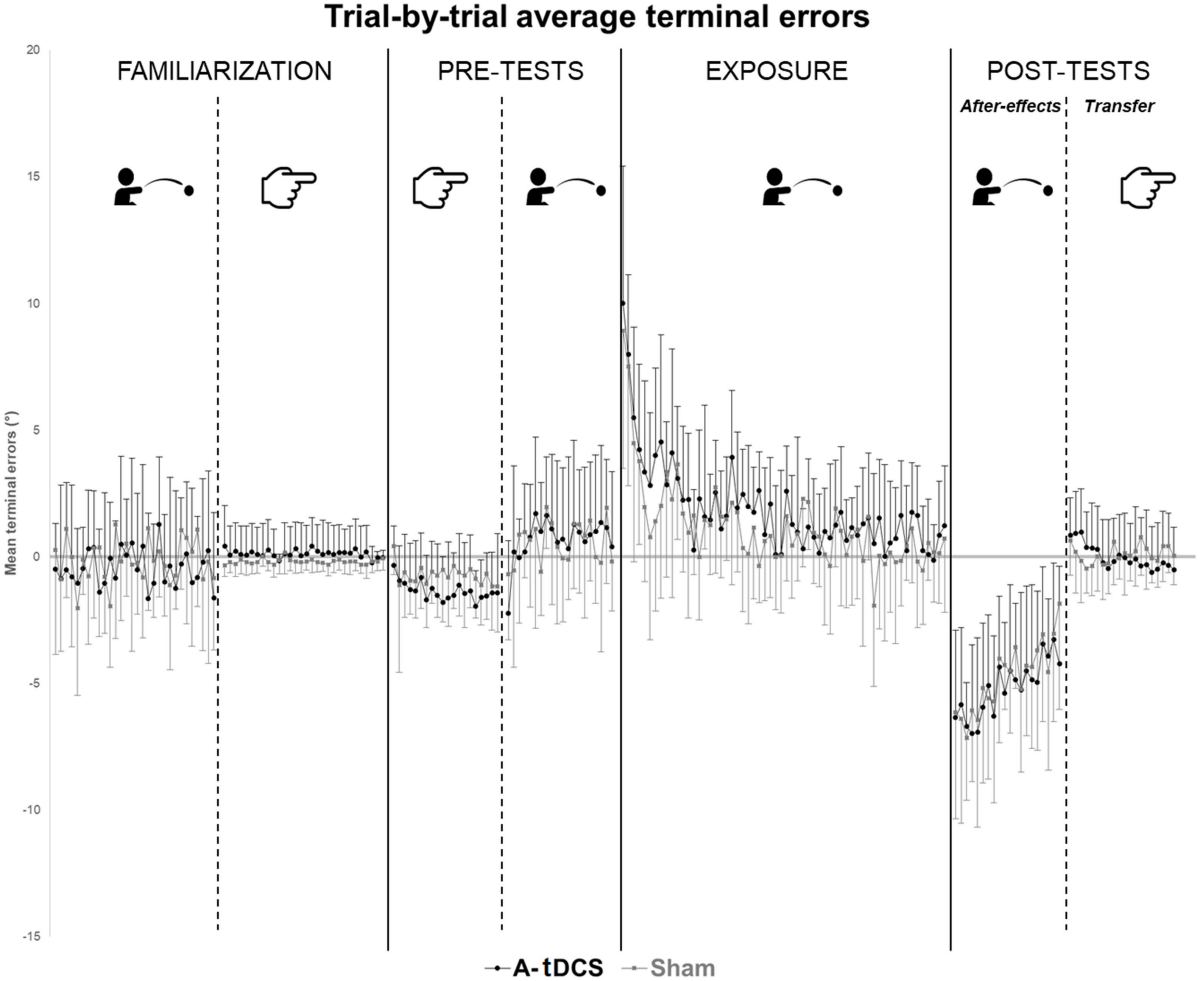
Trial-by-trial average terminal errors. Trial-by-trial average group terminal errors are represented with standard deviations for each group (A-tDCS group in black, Sham group in grey) and for each step of the procedures.

Two participants in the A-tDCS group did not complete the procedure before the time limit (21 min) and were thus removed from the analysis. No participant spontaneously reported awareness of the tDCS condition they were assigned to. Table 1 reports the descriptive statistics, i.e., mean group terminal errors for each phase of the procedure. Figure 2 plots the trial-by-trial mean group terminal errors for the whole experiment.

**Table 1:** Mean group terminal errors during the task phases.

		A-tDCS group	Sham group
Pre-tDCS Familiarization	Throwing	−0.44 ± 1.52	−0.13 ± 0.77
	Pointing	0.11 ± 0.98	−0.19 ± 0.34
Pre-tests	Pointing	−1.35 ± 1.38	−0.73 ± 1.25
	Throwing	0.68 ± 1.84	0.56 ± 1.40
Exposure Throwing	*Trial 1*	10.00 ± 5.42	8.93 ± 5.45
	*Trial 2*	7.98 ± 0.32	7.51 ± 4.69
	*Trial 3*	5.50 ± 3.55	4.49 ± 4.68
	*Trial 4*	4.22 ± 3.38	3.78 ± 3.30
	*Trial 5*	3.35 ± 3.58	1.97 ± 2.95
	*Trial 6*	2.81 ± 2.86	0.78 ± 4.06
	*Trial 7*	4.01 ± 3.42	1.41 ± 3.55
	*Trial 8*	4.53 ± 4.22	2.01 ± 3.64
	*Trial 9*	2.83 ± 2.48	3.35 ± 4.14
	*Trial 10*	4.11 ± 4.10	2.26 ± 3.87
	*Trials 6–10*	3.66 ± 2.79	1.96 ± 1.48
	*Trials 11–20*	1.85 ± 1.32	1.66 ± 0.86
	*Trials 21–30*	1.77 ± 1.29	0.68 ± 0.89
	*Trials 31–40*	1.03 ± 1.17	1.10 ± 0.83
	*Trials 41–50*	0.98 ± 1.23	0.22 ± 0.89
	*Trials 51–60*	0.83 ± 0.89	0.19 ± 1.16
Post-tests	Throwing	−5.18 ± 2.07	−4.71 ± 1.72
	Pointing	−0.01 ± 1.59	0.09 ± 0.92

Values are reported in degrees with standard deviations.

### Familiarization

**Figure 3:**
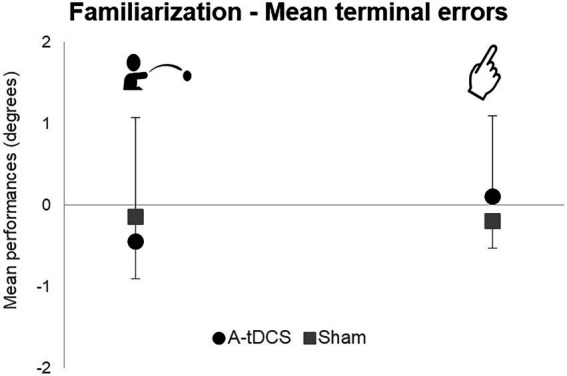
Mean group terminal errors during familiarization. Mean group terminal errors are represented with standard deviations for each group (A-tDCS group in black, Sham group in grey) and for each task (pointing on the right, throwing on the left).

Mean terminal errors during familiarization for the throwing (t(22) = 0.61; *p* = 0.55) and the pointing tasks [*t*(22) = −0.97; *p* = 0.34] did not reveal any difference between groups (Figure 3). Moreover, we observed no difference between groups in mean pointing trajectories orientations during familiarization. Therefore, both groups were comparable during Familiarization.

### Pre-tests

#### Terminal errors

Models analysis revealed no effect of TIME, GROUP and GROUP*TIME interaction on the terminal errors during pre-tests both on pointing and throwing (see Supplementary Table 2 in Supplementary material) indicating that baseline performance was comparable between groups.

#### Pointing trajectories orientations

There was no significant effect of GROUP nor GROUP*TIME interaction on pointing trajectories orientation at initial, intermediate, and terminal directions, showing no effect of the stimulation on these variables during pre-tests (see Supplementary Tables 3–6 in Supplementary material). A significant effect of TIME was observed only for intermediate direction and for the central target [*β* = −0.42, SE = 0.10, *t*(202) = −4.45, *p* < 0.01] indicating that trajectory orientations values tended to decrease through repetition of trials. However, this effect was comparable in the two groups.

### Exposure

#### Terminal errors

Over the whole exposure (60 trials), all participants significantly reduced their errors during the throwing task, as demonstrated by the negative effect of TIME [*β* = −0.06, SE = 0.01, *t*(1407) = −10.12, *p* < 0.01]. This effect was comparable in the two groups as no TIME*GROUP interaction was observed. Noteworthy, the effect of GROUP was close to statistical significance [β = 0.89, SE = 0.47, *t*(46) = 1.88, *p* = 0.06].

During the early phase of exposure (10 first trials of exposure), models showed a significant effect of TIME [*β* = −0.67, SE = 0.12, *t*(213) = −5.63, *p* < 0.01] in the two groups (no GROUP effect and no TIME*GROUP interaction). We found comparable results when considering trials 11 to 60 [TIME: *β* = −0.04, SE = 0.01, *t*(1169) = −5.14, *p* < 0.01] although the slope was reduced. No significant effect of TIME, GROUP, neither their interaction was found when considering the 10 last trials of exposure (51 to 60).

### Post-tests

#### Throwing task: After-effects

The analysis of terminal errors during throwing showed a significant effect of TIME [*β* = 0.20, SE = 0.03, *t*(443) = 6.08, *p* < 0.01] as after-effects reduced with repetition of trials without any group distinction. No GROUP nor GROUP*TIME interaction effect were found.

#### Pointing task: Transfer

##### Terminal errors

We found no effect of TIME or GROUP on pointing terminal error during post-tests. In both groups, average terminal errors were close to zero (mean = −0.01° ± 1.59° for A-tDCS group; mean = 0.09 ± 0.92 in the SHAM group). However, models revealed a significant GROUP*TIME interaction [*β* = −0.08, SE = 0.01, *t*(456) = −5.78, *p* < 0.01]. Longitudinal evolution of terminal errors was not similar across groups: the negative slope was more pronounced in the A-tDCS group meaning that values in this group decreased more rapidly as compared to the sham group.

##### Pointing trajectories orientations

The analysis revealed significant effects of TIME, GROUP and their interaction on trajectory orientations at initial, intermediate and terminal directions of pointing movements (Supplementary Tables 4–6 in Supplementary material).

###### Initial direction

We found a slight but significant effect of TIME (*β* = −0.33, SE = 0.14, t(204) = −2.31, *p* = 0.02) while no effect of GROUP or GROUP*TIME was found. Initial directions were initially oriented to the right and gradually tended to be shifted to the left in both groups upon trials repetition.

###### Intermediate direction

Intermediate direction models showed a significant effect of GROUP*TIME interaction [*β* = −0.27, SE =0.11, *t*(202) = −2.57, *p* = 0.01] indicating that the slopes of transfer curves differed between groups. Intermediate directions were initially slightly shifted to the left and gradually evolved to the right in the SHAM group while they were initially shifted to right and gradually evolved to the left in the A-tDCS group.

###### Terminal direction

We observed a significant effect of GROUP [*β* = 3.64, SE = 1.55, *t*(47) = 2.36, *p* = 0.02] and notable tendencies for the effect of TIME [*β* = 0.28, SE = 0.14, *t*(200) = 1.96, *p* = 0.051], and GROUP*TIME interaction [*β* = −0.37, SE = 0.19, *t*(200) = −1.95, *p* = 0.052] for the central target. Terminal directions globally remained stable over zero (i.e., straight-ahead direction) in the A-tDCS group while they were initially significantly shifted to the left in the SHAM group and gradually evolved to zero upon trials repetition.

We found similar effects for all directions concerning movements toward the right target (see Supplementary Tables 4–6 in Supplementary material, for statistical values).

**Figure 4:**
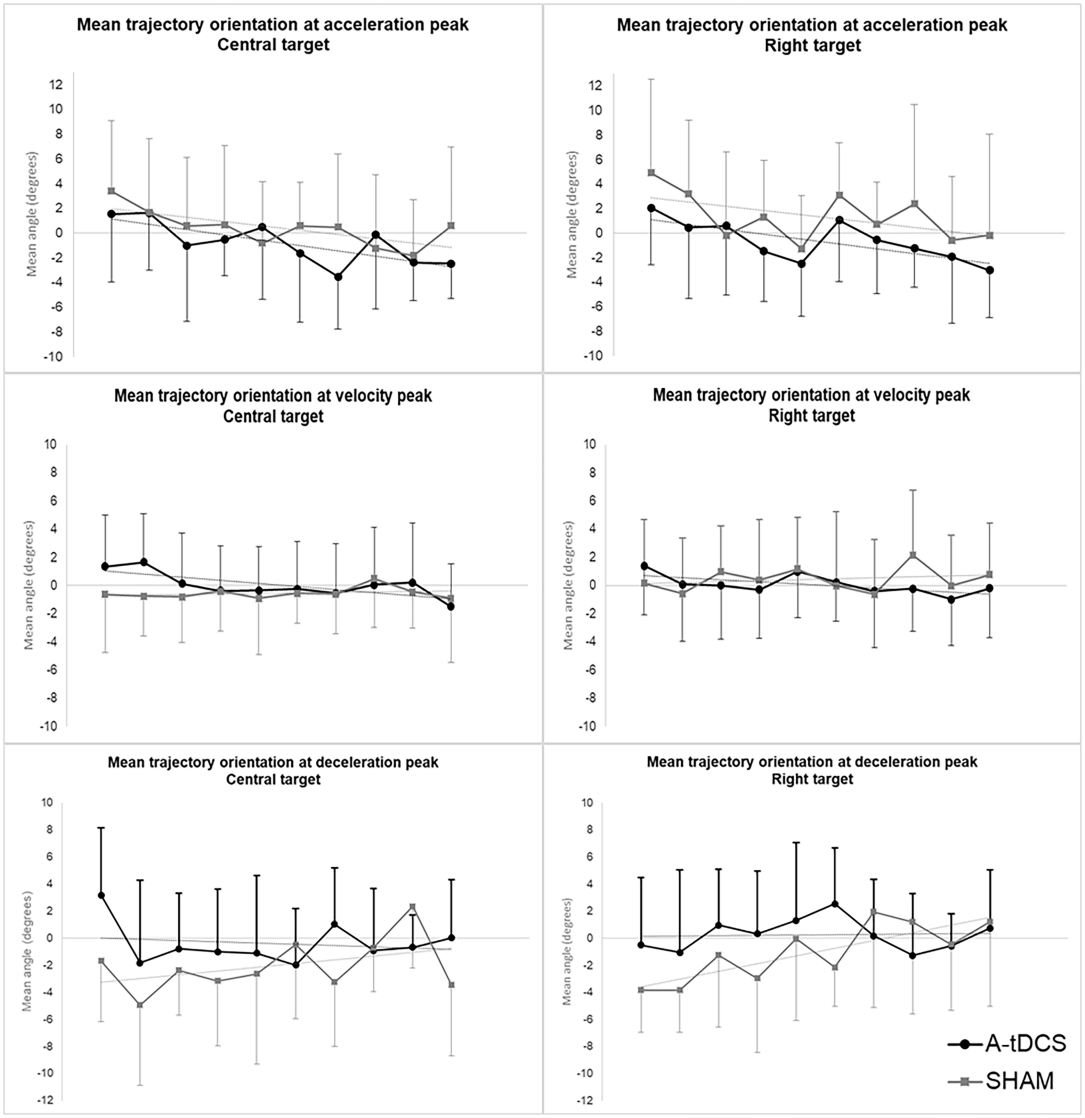
Mean group orientation of pointing trajectories at initial, intermediate, and terminal direction during post-tests. The figure depicts the evolution across trials of mean group orientations of velocity vectors at initial (acceleration peak), intermediate (velocity peak), and terminal (deceleration peak) direction of pointing movements during post-tests for the central target (left) and the right target (right). The A-tDCS group mean values are represented in black; Sham group values are represented in grey. Values are baseline subtracted and plotted with standard deviations. Negative value mean orientation to the left while positive values mean orientation to the right.

Figure 4 displays the longitudinal evolution (trial-by-trial) of mean group trajectory orientations at initial, intermediate, and terminal directions for both targets.

## Discussion

The aim of the study was to investigate the functional role of the cerebellum in after-effects transfer following PA. Anodal tDCS was used to ascertain whether stimulation over the cerebellum was able to boost after-effects transfer from throwing to pointing. Our results demonstrated that tDCS did not significantly affect the overall reduction in terminal errors during exposure (although a notable tendency was observed) and did not impact the amplitude of throwing after-effects. Stimulation did not increase the overall magnitude of transfer from throwing to pointing. However, we found significant stimulation effects on the longitudinal evolution of terminal pointing errors and slight changes in pointing kinematics during post-tests, thus probing a possible implication of the cerebellum in the transfer of after-effects.

### Stimulation effects during exposure and throwing after-effects

A first crucial finding is that anodal tDCS effects on terminal errors during throwing exposure did not reach statistical significance, although we still found a strong tendency for a group effect, suggesting larger errors in the A-tDCS group. A second notable outcome is that both groups demonstrated similar throwing after-effects regardless the stimulation condition, showing that anodal cerebellar tDCS had no effect on the development of throwing after-effects. The results showed that after-effects tended to decrease across repetitions of trials, demonstrating that throwing after-effects were labile. This is reminiscent with our previous investigations of after-effects during throwing prism exposure (Fleury et al., 2020) and with previous studies using throwing during PA procedure (Martin et al., 1996a; Fernández-Ruiz et al., 2000).

Patient studies previously demonstrated the crucial role of the cerebellum in PA, both in error reduction and after-effects (e.g., Weiner et al., 1983; Pisella et al., 2005), by using pointing tasks. This was also reported for throwing prism exposure (Martin et al., 1996b). The activation of cerebellar areas during PA have been reported in neuro-imaging studies and its activation has been confirmed by neuro-stimulation studies from early error reduction to the development of after-effects (Panico et al., 2020). Moreover, previous neuro-stimulation studies using different sensorimotor tasks reported beneficial effects of anodal tDCS on visuomotor adaptation (Galea et al., 2011; Jayaram et al., 2012; Leow et al., 2017). In line with these studies, we expected that anodal tDCS applied to the cerebellum would affect error reduction and after-effects, but our results did not allow to confirm this prediction. However, the strong tendency for a group effect observed during exposure may suggest that the stimulation interfered with some processes at work during exposure. Moreover, as shown by previous studies, comparable behavior when facing the prismatic perturbation and during after-effects assessments does not undoubtedly imply that the same processes took place during exposure: the assessment of inter-task transfer is a method to provide further information about the adaptive processes that were solicited (Fleury et al., 2020). Then, investigating transfer to another task could reveal some tDCS effects that have been partially hidden when looking at terminal errors during exposure and throwing after-effects.

### Stimulation effects during transfer

Although the overall magnitude of transfer was not modified by tDCS, our results showed that stimulation significantly altered the evolution of pointing terminal errors from the middle part of transfer assessment. Indeed, values tended to decrease (i.e., increase in transfer) across trials in the A-tDCS group while remaining stable around zero in the Sham group indicating instead a complete lack of transfer. This pattern of results pointed out a significant effect of stimulation on transfer development. One possible interpretation is that transfer needs more trials during post-tests to be effective. In our setting, the participants only performed 20 trials during transfer. One could argue that some transfer mechanisms occur following exposure and that if the number of pointing was greater, we might have observed a significant effect caused by the stimulation on the overall magnitude of transfer. Further studies using more trials to assess transfer could better investigate the issue.

Besides, we also observed significant alterations in some kinematics parameters of pointing caused by the stimulation during transfer assessment. The initial direction of pointing movements significantly and negatively evolved across trial repetition during pointing post-tests, i.e., the initial part of the movement tended to be gradually shifted toward the left upon trials repetition. This effect of time was comparable in both groups, regardless stimulation condition. When considering intermediate direction, results demonstrated that the trial-by-trial evolution of trajectory orientation at the peak of velocity (i.e., middle part) was significantly different between groups, showing a slight increase for the SHAM group and a slight decrease over time for the A-tDCS group. This means that participants who received the stimulation tended to slightly and gradually shift their pointing trajectory at the middle part toward the left across trials repetition during transfer assessment, while the middle direction of trajectories were initially slightly shifted to the left and evolved to the right in the SHAM group. Concerning the terminal part of pointing trajectories, we found a strong tendency for a GROUP*TIME interaction indicating that terminal orientations of trajectories were globally stable around zero (i.e., straight-ahead direction) over time in the A-tDCS group while in they were not in the SHAM: terminal parts of reaching were initially more oriented to the left end were corrected over time to reach values around zero. Related to this, we also found that mean trajectory terminal orientation was overall significantly lower (i.e., more oriented to the left) in the SHAM group than in the A-tDCS group.

Taken together, these results indicate that trajectories kinematics and their evolution during transfer assessment differed between groups which is likely due to the stimulation. The differences observed can be interpreted as following: both groups showed comparable behavior in the early part of trajectories (shift to the right) that could be related to use-dependent modification of pointing arising from repetition of movement deviated to the right during exposure (McDougle et al., 2016). However, differences arising from the middle part and upon trials repetition may indicate a gradual renormalization of trajectories in the SHAM group while trajectories remained perturbed in the A-tDCS group. Therefore, the stable terminal directions observed in the A-tDCS could reflect the absence of corrective adjustments during the terminal part of the pointing movements suggesting a lasting trace of adaptation related to transfer. Therefore, the stimulation had an effect mainly from the middle part of the trajectory, i.e., the corrective part of reaching. These findings are compatible with results from our previous tDCS study (Fleury et al., 2021). In this work, we demonstrated that the corrective part of pointing trajectories during after-effects assessment differed in participants who received a cathodal cerebellar stimulation versus participants who received a placebo stimulation. These evidence suggest an effect of the stimulation on the ability to (1) detect a mismatch between the expected and the actual proprioceptive feedback during reaching and (2) to implement this sensory prediction error online to correct the movement before its end. Both these functions are known to rely on the cerebellum (Popa and Ebner, 2018). Therefore, this could support the role of the cerebellum in processes underlying online correction during PA as already discussed in previous work (Panico et al., 2018b). However, such effects of the stimulation were observable through kinematics while they did not lead to a significant change in pointing accuracy that could perhaps has been observed using more trials in transfer assessment.

### General interpretation and limitations

These effects, together with the strong tendency observed during throwing exposure, indicate that stimulation have elicited some effects on transfer processes. The influence of anodal tDCS on adaptive processes at work during exposure may have been partially hidden when looking at usual measures of performance (error reduction and throwing after-effects) but uncovered by the analysis of after-effects transfer on pointing, i.e., differences were mainly observed during transfer assessment. Although we cannot ascertain the expected boosting effect of tDCS, the present results add probes in favor of the implication of the cerebellum in PA after-effects transfer. In fact, interfering with this area by means of noninvasive brain stimulation impacted pointing performance during transfer, thus supporting results from Fleury et al. (2021).

Nonetheless, it is worth mentioning that the present findings could not unquestionably attest that anodal tDCS positively modulated after-effects transfer because the stimulation did not affect the overall magnitude of transfer from throwing to pointing. Several lines of arguments can be raised to discuss this aspect.

Previous evidence suggested that the practice of a non-mastered task during exposure to prisms modulated the nature of processes at work to face the perturbation (Fleury et al., 2020). Here, participants were novice in throwing and might have solicited processes that did not lead to transferable after-effects. We may speculate that during throwing prism exposure, novice participants might rely more deeply on strategic processes of error reduction, i.e., recalibration, as compared to pointing prism exposure, while the effect of realignment might have been reduced. This is compatible with evidence that different cerebellar areas participate in recalibration and realignment (Luauté et al., 2009; Chapman et al., 2010; Küper et al., 2014; Panico et al., 2020, 2021). Conversely, in participants with expertise in throwing, a more balanced relationship between recalibration and realignment could be observed, possibly allowing them to develop effective transfer from throwing to pointing (Fleury et al., 2020). Thus, we could hypothesize that a certain level of expertise on the task practiced during exposure is needed to obtain effective tDCS boosting effects on transfer. To test whether anodal stimulation only boost high level performance, future studies should compare a group of novices to a group of experts in throwing. This interpretative framework is in line with a former study that reported task-dependent effects of anodal tDCS (although applied to the primary motor cortex) across various motor learning tasks (Karok et al., 2017). In this latter study, task characteristics modulated the neural state of the different brain regions and caused differential stimulation effects. Therefore, the state of cerebellar regions through our throwing PA procedure involving novice participants in throwing could have attenuated the effects of the tDCS. We therefore speculate that the contribution of cerebellar mechanisms would have been greater in participants with a higher degree of expertise in throwing. Another interesting aspect would be to compare the effect of anodal cerebellar stimulation in groups of different ages. Sensorimotor adaptation declines with age (Heuer and Hegele, 2008; Vandevoorde and Orban de Xivry, 2019), and neuromodulation has been shown to be effective in restoring abnormal sensorimotor adaptation in older adults (Zimerman and Hummel, 2010; Hardwick and Celnik, 2014; Panouillères et al., 2015). Therefore, one may expect more pronounced positive effects of anodal cerebellar tDCS during prism adaptation in older adults compared to young, as the initial sensorimotor capacities is altered with aging but might be restored with neuromodulation.

Finally, it is also worth discussing the directionality of the tDCS effects on transfer. While the evolution of pointing terminal errors indicated that tDCS tended to increase transfer magnitude, the kinematic analysis showed differential modifications of pointing trajectories and did not convey in identifying a potential enhancement of after-effects transfer. In addition, the tendency we observed during exposure was not compatible with any possible facilitating effect, as errors were larger in the A-tDCS group. Consequently, it is not straight-forward to determine whether anodal tDCS during throwing PA would boost adaptation or should, conversely, impair it. Bortoletto et al. (2015) reported conflicting effects of anodal tDCS when interacting with varying motor tasks. They showed unexpected detrimental effects of the tDCS when combined with a learning task, thus reporting non-additive mechanisms between two sources of induced-plasticity, i.e., a possible negative interference between learning and tDCS. This is supported by the fact that no evidence is available concerning the polarity-dependent effects of cerebellar tDCS as reported in a previous meta-analysis (Summers et al., 2016). Therefore, further investigation is needed to test the additive effects of anodal tDCS and PA procedure using throwing or another motor task during exposure.

Important aspects could limit the interpretability of the results and need to be emphasized. The sample size is modest, which limits the power of the study. It is especially crucial given the high inter-individual variability in throwing performance and possible variability in responsiveness to the stimulation. Therefore, the present result might be interpreted with cautious. A higher number of subjects would allow to undertake an individual-centered approach by looking for individual features that could explain different behavioral patterns of transfer (e.g., Renault et al., 2018). On the other hand, computational modeling approaches could also bring additional and crucial information about lobules-specific electric field distribution in a subject-specific manner (e.g., Rezaee and Dutta, 2019). Such approach would allow to optimize the placement of the active electrode in order to maximize the current density through the targeted area while considering individual anatomical features (Batsikadze et al., 2019; Ponce et al., 2021). While the present study is a purely behavioral investigation, further work using computational modeling methods would help to reduce inter-individual variability in the responsiveness to the stimulation and offer to go deeper in the mechanistic explanations related to the efficacy of the stimulation on PA and transfer. In addition, as conventional tDCS is limited in terms of spatial resolution, modern high-definition tDCS montages might be useful to selectively target specific cerebellar regions and limit the current spreading (Panico et al., 2022).

### Opened perspectives from a network approach

The cerebellum comprises distinct and overlapping functional zones, i.e., a primary sensorimotor zone (lobules V, VI, and VIII) and a supramodal zone (lobules VIIa, Crus I and II) which are related to specific patterns of connectivity with other areas of the brain (e.g., motor and somatosensory cortices for the sensorimotor zone and prefrontal and posterior-parietal cortices for the supramodal zone; O’Reilly et al., 2010). Therefore, considering a network approach of PA, it would be relevant to investigate the precise activation of this distinct zones and their overlapping connectivity maps during the transfer of after-effects using neuro-imaging such as fMRI (Bultitude et al., 2017) to record task-related functional brain connectivity with a high spatial resolution (Rogers et al., 2007).

Interesting perspectives also lie in the stimulation and the role of other areas in transfer, in particular the primary motor cortex (M1). Indeed, studies on motor learning and consolidation showed that M1 plays an important role in acquisition, consolidation and retention of motor memories (e.g., Galea et al., 2011; Karok et al., 2017). This has also been highlighted in PA studies, showing that anodal stimulation of M1 affected after-effect development, stabilizing both sensorimotor and cognitive prism after-effects (O’shea et al., 2017; Panico et al., 2017). Recently, Panico et al. (2021), put forward a substantial role of M1 in PA. We might speculate that the cerebellum provides the adaptive mechanisms to develop transfer but then a consolidation phase in M1 is needed to fully observe transfer. Then, further tDCS studies could test for the implication of M1 in transfer in a longitudinal perspective with assessments during different time windows following PA, or using increasing number of trials during post-tests. It is also likely that reciprocal connections between M1 and the cerebellum are responsible for the full development of transfer. This interpretation goes against the idea of a clear-cut separation of the involvement of distinct brain areas in PA processes as depicted in classical models (Clower et al., 1996; Redding et al., 2005; Danckert et al., 2008; Luauté et al., 2009; Chapman et al., 2010; Küper et al., 2014) and is rather in favor of dynamic interconnections between several areas. This would be in line with previous stimulation studies probing the existence of a cerebello-parietal circuitry involved in PA using multiple-sites stimulation targeting both areas either with opposite (bi-cephalic stimulation; Panico et al., 2018a) or concurrent polarities (using HD-tDCS; Panico et al., 2022). These findings were supported by neuroimaging insights that highlighted the modulation of a cerebello-parieto-parahippocampal network during PA (Schintu et al., 2020). Therefore, one may hypothesize that M1 also plays a role within this complex and dynamic PA network, in particular concerning the consolidation and the transfer of after-effects. Further multiple-sites HD-tDCS or multifocal stimulation (as a previous work in skill learning; Wessel et al., 2021) studies should be conducted to investigate the role of the M1-cerebellum interplay in transfer.

### Conclusion

To conclude, although we could not ascertain the boosting effect of anodal tDCS on transfer from throwing to pointing in novice, the present results add a piece of evidence to the contribution of the cerebellum in the development of inter-task transfer in PA. Further studies should test the possibility to use anodal stimulation to modulate the magnitude of inter-task transfer with expert throwers or using other mastered tasks during exposure as well as the potential role of M1 and its interaction with the Cerebellum in a complex circuitry responsible for the development of transfer. Still, the present study represents an additional probe that the assessment of inter-task transfer brings supplemental information regarding the nature of adaptive processes at work to face a prismatic shift, beyond the classical measure of after-effects. In addition, this study emphasizes the interest of analyzing after-effects and transfer of after-effects across trial-by-trial evolution rather than averaging all post-tests trials. Overall, our conclusions pave the way for further pieces of work investigating the mechanisms and the underlying neural substrates involved in the transfer of after-effects in Prism Adaptation.

## Data availability statement

The raw data supporting the conclusions of this article will be made available by the authors, without undue reservation.

## Ethics statement

The studies involving human participants were reviewed and approved by “CPP SUD-EST IV,” ID-RCB: 2010-A01180-39. The patients/participants provided their written informed consent to participate in this study.

## Author contributions

LF, FP, and YR contributed to conception and design of the study. LF performed data collection, formal analysis, statistical analysis, and wrote the first draft of the manuscript. FP assisted data collection, statistical analyses, and substantially revised the manuscript. AF substantially contributed to the statistical analyses. PR and LD assisted data collection and revised the manuscript. CC and YR supervised the study and revised the manuscript. SJ-C revised the manuscript. All authors contributed to the article and approved the submitted version.

## Funding

Open access funding was provided by the École Polytechnique Fédérale de Lausanne.

## Conflict of interest

The authors declare that the research was conducted in the absence of any commercial or financial relationships that could be construed as a potential conflict of interest.

## Publisher’s note

All claims expressed in this article are solely those of the authors and do not necessarily represent those of their affiliated organizations, or those of the publisher, the editors and the reviewers. Any product that may be evaluated in this article, or claim that may be made by its manufacturer, is not guaranteed or endorsed by the publisher.
