# Erratum: Does anodal cerebellar tDCS boost transfer of after-effects from throwing to pointing during prism adaptation?

**DOI:** 10.3389/fpsyg.2023.1176485

**Published:** 2023-03-09

**Authors:** Frontiers Production Office

**Affiliations:** Frontiers Media SA, Lausanne, Switzerland

**Keywords:** prism adaptation (PA), transfer, cerebellum, anodal tDCS, sensorimotor plasticity

An omission to the funding section of the original article was made in error. The following sentence has been added: “Open access funding was provided by the École Polytechnique Fédérale de Lausanne.”

The publisher apologizes for this mistake. The original article has been updated.

