# Supplementary material for: Does anodal cerebellar tDCS boost transfer of after-effects from throwing to pointing during prism adaptation?
Source: Front Psychol. 2022 Sep 27;13:909565. doi: 10.3389/fpsyg.2022.909565 (PMC9552335; doi:10.3389/fpsyg.2022.909565)
Supplement: Supplementary file 1 [file Data_Sheet_1.PDF]

Supplementary Materials

Deviations - Linear Mixed Models Analysis

		Fixed Effects						Random effects / Model parameters		
		Estimate	Std Error	df	t	p			Variance	Std Err
**Fam Pointing**	Intercept	-0.19	0.22	25.42	-0.85	0.40		Intercept (Id)	0.52	0.72
	Time	0.00	0.00	688.00	0.01	0.99		Residual	0.16	0.40
	Group	0.33	0.30	25.46	1.10	0.28		logLik	-419.30	ICC
	Group*Time	0.00	0.00	688.00	-0.61	0.55		Nb Obs	712.00	0.76
**Fam Throwing**	Intercept	-0.25	0.44	53.35	-0.57	0.57		Intercept (Id)	1.16	1.08
	Time	0.01	0.02	688.12	0.46	0.65		Residual	7.32	2.71
	Group	-0.20	0.59	53.29	-0.33	0.74		logLik	-1739.40	ICC
	Group*Time	-0.01	0.02	688.15	-0.33	0.74		Nb Obs	712.00	0.14
**Pre-tests Throwing**	Intercept	0.34	0.55	39.86	0.63	0.53		Intercept (Id)	2.25	1.50
	Time	0.02	0.03	447.93	0.84	0.40		Residual	5.52	2.35
	Group	-0.33	0.74	39.98	-0.45	0.66		logLik	-1099.30	ICC
	Group*Time	0.05	0.04	448.03	1.29	0.20		Nb Obs	472.00	0.29
**Pre-tests Pointing**	Intercept	-0.51	0.41	31.10	-1.25	0.22		Intercept (Id)	1.53	1.24
	Time	-0.02	0.01	454.01	-1.55	0.12		Residual	1.62	1.27
	Group	-0.49	0.55	31.05	-0.89	0.38		logLik	-829.80	ICC
	Group*Time	-0.01	0.02	454.01	-0.67	0.50		Nb Obs	478.00	0.48
**Exposure Throwing 1-60**	Intercept	3.14	0.35	46.86	8.98	0.00	***	Intercept (Id)	0.83	0.91
	Time	-0.06	0.01	1407.00	-10.12	< 2e-16	***	Residual	7.79	2.79
	Group	0.89	0.47	46.62	1.88	0.07	.	logLik	-3522.90	ICC
	Group*Time	-0.01	0.01	1407.00	-0.96	0.34		Nb Obs	1431.00	0.10
**Post-tests Throwing**	Intercept	-6.60	0.64	41.36	-10.40	0.00	***	Intercept (Id)	3.00	1.73
	Time	0.20	0.03	443.24	6.08	0.00	***	Residual	7.26	2.70
	Group	-0.12	0.86	40.86	-0.14	0.89		logLik	-1152.00	ICC
	Group*Time	-0.04	0.04	443.20	-0.81	0.42		Nb Obs	467.00	0.29
**Post-tests Pointing**	Intercept	-0.04	0.40	27.46	-0.11	0.91		Intercept (Id)	1.58	1.26
	Time	0.02	0.01	456.00	1.41	0.16		Residual	0.84	0.91
	Group	0.68	0.54	27.46	1.27	0.22		logLik	-681.70	ICC
	Group*Time	-0.08	0.01	456.00	-5.78	0.00	***	Nb Obs	480.00	0.65

Table 2 - Linear Mixed Models Analysis results - Deviations. *Effects of TIME, GROUP and GROUP*TIME on terminal errors at the different steps of the experimental procedure.*

Pointing trajectories orientations - Descriptive statistics

		Mean orientation at initial direction		Mean orientation at intermediate direction		Mean orientation at final direction	
		ctDCS group	***Sham group***	***ctDCS group***	***Sham group***	***ctDCS group***	***Sham group***
**Familiarization**	*Pointing*	-3.41 ± 6.59	-5.01 ± 5.98	3.86 ± 2.17	2.60 ± 1.74	3.28 ± 7.48	4.47 ± 7.00
**Pre-tests**	*Central*	-7.17 ± 9.31	-9.49 ± 8.22	2.11 ± 2.35	2.00 ± 4.14	4.29 ± 5.73	5.91 ± 5.92
	Right	-7.99 ± 8.75	-11.48 ± 6.79	6.61 ± 3.40	6.20 ± 3.68	14.19 ± 4.15	17.19 ± 7.72
**Post-tests**	*Central*	0.47 ± 4.96	2.26 ± 6.97	0.11 ± 2.62	-0.44 ± 2.61	0.48 ± 4.19	-1.72 ± 4.30
	Right	-0.04 ± 3.01	2.21 ± 5.83	-0.40 ± 3.20	0.27 ± 3.25	1.36 ± 2.98	-1.32 ± 4.73

Table 3 – Pointing trajectories orientations at initial (acceleration peak), intermediate (velocity peak) and final (deceleration peak) directions. *Values are reported in degrees with standard deviations.*

Pointing trajectories orientations at initial direction - Linear Mixed Models Analysis

		Fixed Effects						Random effects / Model parameters		
		Estimate	Std Error	df	t	p			Variance	Std Err
Fam Pointing	Intercept	-3.91	1.82	25.56	-2.15	0.042	*	Intercept (Id)	35.09	5.924
	Time	-0.11	0.02	687.02	-4.72	0.000	***	Residual	12	3.464
	Group	1.11	2.48	25.55	0.45	0.658		logLik	-1946	ICC
	**Group*Time**	**0.06**	**0.03**	**687.01**	**2.11**	**0.035**	*****	Nb Obs	711	0.75
Pre-tests Pointing central target	Intercept	-8.14	2.65	27.16	-3.07	0.005	**	Intercept (Id)	69.91	8.361
	**Time**	**-0.29**	**0.15**	**200.44**	**-1.92**	**0.056**	**.**	Residual	18.59	4.311
	Group	1.71	3.59	27.00	0.48	0.637		logLik	-687.8	ICC
	Group*Time	0.12	0.20	200.18	0.58	0.566		Nb Obs	224	0.79
Pre-tests Pointing right target	Intercept	-11.59	2.40	27.75	-4.83	0.000	***	Intercept (Id)	56.91	7.544
	Time	0.04	0.14	204.19	0.27	0.791		Residual	15.78	3.973
	Group	4.75	3.25	27.40	1.46	0.155		logLik	-680.5	ICC
	Group*Time	-0.29	0.18	204.04	-1.55	0.123		Nb Obs	228	0.78
Post-tests Pointing central target	Intercept	3.17	1.85	48.16	1.72	0.092	.	Intercept (Id)	21.63	4.651
	Time	-0.22	0.23	216.00	-0.97	0.333		Residual	46.09	6.789
	Group	-0.92	2.51	48.16	-0.37	0.716		logLik	-821.1	ICC
	Group*Time	-0.33	0.31	216.00	-1.07	0.287		Nb Obs	240	0.32
Post-tests Pointing right target	Intercept	3.58	1.66	30.89	2.15	0.039	*	Intercept (Id)	25.34	5.034
	Time	-0.19	0.13	216.00	-1.52	0.131		Residual	14.82	3.85
	Group	-2.46	2.26	30.89	-1.09	0.286		logLik	-698.8	ICC
	Group*Time	-0.19	0.17	216.00	-1.11	0.269		Nb Obs	240	0.63

Table 4 - Linear Mixed Models Analysis results – pointing trajectories orientations at initial direction. *Effects of TIME, GROUP and GROUP*TIME on pointing trajectories orientations at initial direction and at the different steps of the experimental procedure.*

Pointing trajectories orientations at intermediate direction - Linear Mixed Models Analysis

		Fixed Effects						Random effects / Model parameters		
		Estimate	Std Error	df	t	p			Variance	Std Err
Fam Pointing	Intercept	3.09	0.62	31.43	5.00	0.000	***	Intercept (Id)	3.48	1.87
	Time	-0.04	0.02	688.04	-2.59	0.010	**	Residual	5.67	2.38
	Group	0.35	0.84	31.39	0.42	0.678		logLik	-1663.60	ICC
	**Group*Time**	**0.07**	**0.02**	**688.00**	**3.30**	**0.001**	******	Nb Obs	712.00	0.38
Pre-tests Pointing central target	Intercept	3.83	1.25	30.41	3.05	0.005	**	Intercept (Id)	14.51	3.81
	**Time**	**-0.42**	**0.10**	**202.14**	**-4.45**	**0.000**	*******	Residual	7.34	2.71
	Group	-1.53	1.70	30.23	-0.90	0.375		logLik	-578.80	ICC
	Group*Time	0.18	0.13	201.75	1.43	0.156		Nb Obs	225.00	0.66
Pre-tests Pointing right target	Intercept	6.52	1.42	28.79	4.58	0.000	***	Intercept (Id)	19.61	4.43
	Time	-0.06	0.09	204.52	-0.74	0.462		Residual	6.44	2.54
	Group	-0.85	1.92	28.37	-0.44	0.662		logLik	-576.40	ICC
	Group*Time	-0.07	0.12	204.35	-0.61	0.541		Nb Obs	228.00	0.75
Post-tests Pointing central target	Intercept	-0.72	0.86	35.42	-0.85	0.404		Intercept (Id)	6.08	2.47
	Time	0.06	0.08	203.15	0.72	0.476		Residual	4.94	2.22
	Group	1.67	1.16	34.54	1.45	0.158		logLik	-531.30	ICC
	Group*Time	-0.27	0.10	202.73	-2.53	0.012	*	Nb Obs	226.00	0.55
Post-tests Pointing right target	Intercept	0.22	0.96	31.31	0.23	0.816		Intercept (Id)	8.30	2.88
	Time	0.08	0.08	204.69	1.09	0.278		Residual	5.06	2.25
	Group	0.48	1.30	31.13	0.37	0.717		logLik	-541.80	ICC
	Group*Time	-0.23	0.10	204.42	-2.24	0.026	*	Nb Obs	228.00	0.62

Table 5 - Linear Mixed Models Analysis results – pointing trajectories orientations at intermediate direction. *Effects of TIME, GROUP and GROUP*TIME pointing trajectories orientations at intermediate direction and at the different steps of the experimental procedure.*

Pointing trajectories orientations at terminal direction - Linear Mixed Models Analysis

		Fixed Effects						Random effects / Model parameters		
		Estimate	Std Error	df	t	p			Variance	Std Err
**Fam Pointing**	Intercept	3.34	2.10	26.63	1.59	0.124		Intercept (Id)	45.34	6.73
	Time	0.08	0.03	685.04	2.37	0.018	*	Residual	26.12	5.11
	Group	-0.52	2.86	26.64	-0.18	0.856		logLik	-2210.10	ICC
	Group*Time	-0.04	0.04	685.03	-0.96	0.336		Nb Obs	709.00	0.63
**Pre-tests Poiting central target**	Intercept	6.26	1.79	34.92	3.50	0.001	**	Intercept (Id)	26.53	5.15
	Time	-0.10	0.17	202.65	-0.57	0.568		Residual	22.97	4.79
	Group	-0.75	2.42	34.65	-0.31	0.758		logLik	-701.20	ICC
	Group*Time	-0.25	0.23	202.14	-1.12	0.265		Nb Obs	225.00	0.54
**Pre-tests Pointing right target**	Intercept	16.91	2.00	36.67	8.46	0.000	***	Intercept (Id)	32.14	5.67
	Time	0.05	0.19	205.01	0.24	0.808		Residual	29.24	5.41
	Group	-2.67	2.69	35.67	-0.99	0.329		logLik	-737.40	ICC
	Group*Time	-0.01	0.25	204.64	-0.05	0.961		Nb Obs	228.00	0.52
**Post-tests Pointing central target**	Intercept	-3.11	1.12	70.07	-2.78	0.007	**	Intercept (Id)	5.21	2.28
	Time	0.34	0.16	203.63	2.08	0.039	*	Residual	21.26	4.61
	Group	3.04	1.49	66.65	2.04	0.046	*	logLik	-680.40	ICC
	Group*Time	-0.44	0.22	202.54	-2.01	0.046	*	Nb Obs	226.00	0.20
**Post-tests Pointing right target**	Intercept	-3.44	1.23	38.61	-2.79	0.008	**	Intercept (Id)	11.67	3.42
	Time	0.49	0.13	205.40	3.80	0.000	***	Residual	13.94	3.73
	Group	3.39	1.67	38.28	2.03	0.049	*	logLik	-650.00	ICC
	Group*Time	-0.39	0.17	204.98	-2.28	0.024	*	Nb Obs	228.00	0.46

Table 6 - Linear Mixed Models Analysis results – pointing trajectories orientations at terminal direction. *Effects of TIME, GROUP and GROUP*TIME pointing trajectories orientations at terminal direction and at the different steps of the experimental procedure.*
